# Azobenzene as Multi-Targeted Scaffold in Medicinal Chemistry

**DOI:** 10.3390/molecules29245872

**Published:** 2024-12-12

**Authors:** Barbara De Filippis, Alice Della Valle, Alessandra Ammazzalorso, Cristina Maccallini, Giuseppe Tesse, Rosa Amoroso, Adriano Mollica, Letizia Giampietro

**Affiliations:** Department of Pharmacy, University “G. d’Annunzio”, 66100 Chieti, Italy; barbara.defilippis@unich.it (B.D.F.); alice.dellavalle@unich.it (A.D.V.); alessandra.ammazzalorso@unich.it (A.A.); cristina.maccallini@unich.it (C.M.); giuseppe.tesse@unich.it (G.T.); rosa.amoroso@unich.it (R.A.); adriano.mollica@unich.it (A.M.)

**Keywords:** azobenzene, azo-compounds, multi-target, phenyldiazenyl, phenylazo moiety

## Abstract

The discovery of a multi-target scaffold in medicinal chemistry is an important goal for the development of new drugs with different biological effects. Azobenzene is one of the frameworks in medicinal chemistry used for its simple synthetic methods and for the possibility to obtain a great variety of derivatives by simple chemical modifications or substitutions. Phenyldiazenyl-containing compounds show a wide spectrum of pharmacological activities, such as antimicrobial, anti-inflammatory, anti-neurodegenerative, anti-cancer, and anti-enzymatic. The aim of this review is to highlight the importance of azobenzene as a scaffold in medicinal chemistry, with particular attention to the chemical modifications and structure–activity relationships (SARs). This review emphasizes the main therapeutic applications of phenyldiazenyl derivatives, with a particular focus on structural modification and its influence on activity, with the aim of inspiring medicinal chemists to obtain new, increasingly powerful azobenzenes useful in therapy.

## 1. Introduction

Azo derivatives are a class of molecules containing at least one R-N=N-R′ functional group in which R and R′ could be an alkyl or aryl group, giving two distinct classes of azo compounds. Aliphatic azo compounds are mostly colorless and less stable than arylazo compounds. Some alkyl azo compounds act as radical initiators after cleavage of the C-N bond by irradiation or at high temperatures. Aromatic azo compounds are more common and highly stable due to the presence of aryl groups on both sides of the -N=N- group that extend the delocalized system [1]. A greater conjugation of the π system allows for major absorption capacity in the visible range (400–700 nm) and induces a more intense color of azo compounds. The nature and position of the substituents on the aromatic rings determine the color of phenylazo compounds. In this class, the azo moiety (-N=N-) is conjugated with two identical or different mono- or polycyclic aromatic rings. They have no natural origin and were synthetized by Bismarck in the 1860s [2] and then used as dyes in the textile industry.

Different synthetic strategies can be applied to obtain azo compounds, including the oxidation of aromatic amines, the reduction of aromatic compounds with nitroso groups, the coupling reaction of arylamines with nitroso compounds, the oxidation of hydrazines, the reduction of azoxybenzene derivatives, and the azo-coupling of diazonium salts, as reported by Hamon et al. [3].

**Figure 1:**
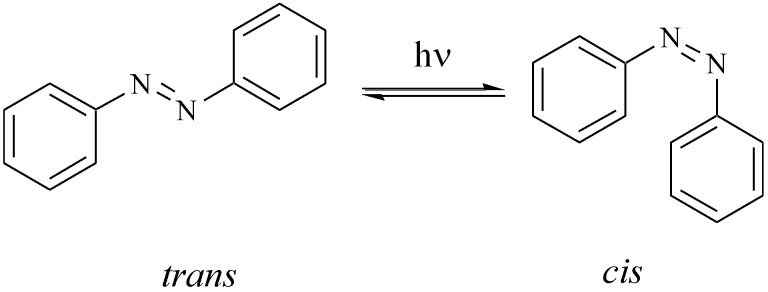
Photoisomerization of azobenzene.

Azo derivatives can exist in two different configurations, the *trans* or “*E*” form and the *cis* or “*Z*” form. To pass from the more stable *trans* form to the *cis* form, azobenzene requires energy of around 50 kJ/mol. After exposure to external stimuli such as light or heat, a simple molecule such as azobenzene can be induced to the *cis–trans* interchange. The conformation of compounds changes without bond breaking. For example, upon exposure to light of a certain wavelength (350 nm), the *trans* form photoisomerizes to the *cis* form and, upon thermal and/or phytochemical treatment, switches back the *cis* configuration into the *trans* one (Figure 1).

**Figure 2:**
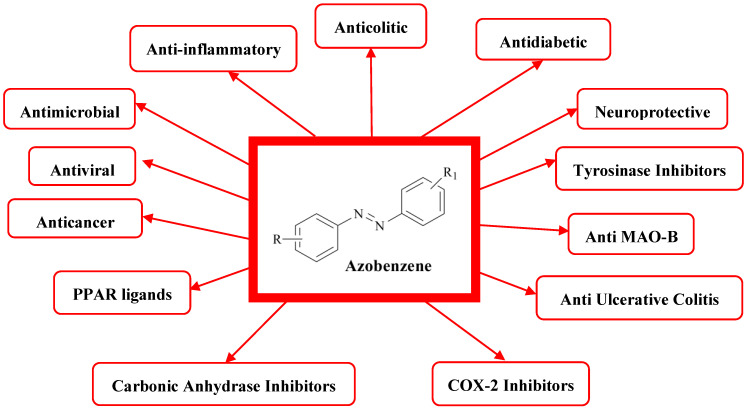
Main pharmacological activities of azobenzene derivatives.

The properties of the two isoforms are influenced by the different positions and types of substituents, which also determine different pharmacological properties. With ^1^H-NMR spectroscopy, it is possible to establish the geometry of the aromatic rings because different fields can be distinguished for *cis* and *trans* signals. Considering the importance of azo derivatives in medicinal chemistry and the modification of their activities depending on the substitution of azobenzene moiety [4], this review summarizes the main biological activities of compounds containing phenylazo moiety reported over the last few years (Figure 2). Moreover, several modifications of the azobenzene scaffold are described in order to define the structural requirements for optimal biological profiles.

## 2. Synthesis

**Scheme 1:**
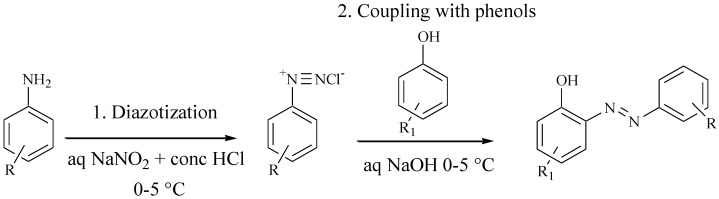
Synthesis of generic phenyl azo compounds by diazotization and coupling.

Phenydiazenyl compounds can be prepared by different synthetic strategies [3]. The simplest is the formation of diazonium salt by diazotization and subsequent coupling with a phenolic component (Scheme 1) [1]. The starting aromatic amine is dissolved in water with concentrated HCl; this solution is cooled in ice because diazonium salts are usually unstable at room temperature. An aqueous cold solution of sodium nitrite is added under stirring. After a range of time that can be from 2 min up to one hour, this solution is added to an aqueous sodium hydroxide solution of a generic phenolic compound and stirred at 0–5 °C. The formation of a solid-colored azo compound is usually observed after 4 h, which is then filtered and dried in vacuum.

## 3. Biological Activities

Compounds containing an azo linker have been shown to have numerous properties in, for example, antiseptic, antimicrobial, antidiabetic, antineoplastic, transmissible spongiform encephalopathic, antiulcerative, antioxidant, analgesic, anti-inflammatory, antiviral, antitubercular, and antitumor activities. They are also involved in biological reactions such as the inhibition of DNA, RNA and protein synthesis, carcinogenesis, and nitrogen fixation [5]. In view of the diverse pharmacological activities of azobenzene derivatives, the main ones are reported below.

### 3.1. Anticolitic Properties

A prodrug is a pharmacologically inactive entity that is converted into its active form by a chemical or enzymatic method. The active drug and the nontoxic portion are released by internal or external stimuli at a targeted site within the body. Due to the cleavable character of the azo bond, azo compounds can act as drug carriers, as shown in the review by Mutlu et al. [6].

**Figure 3:**
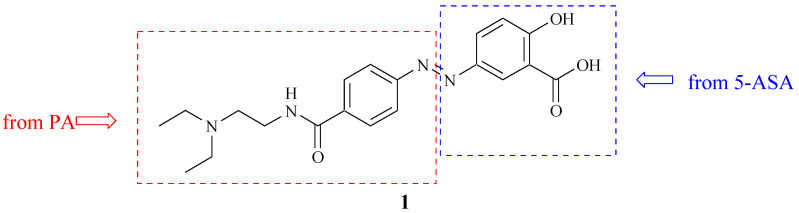
Chemical structure of 5-ASA-azo-PA (**1**).

Azo prodrugs are involved in the treatment of colon diseases for the release of specific amines. Kim et al. studied the application of azo compounds as prodrugs in inflammatory bowel disease (IBD) [7]. This disease is a chronic inflammation of the gut, usually referred to as ulcerative colitis (UC) or Crohn’s disease (CD). To improve the therapeutic activity of 5-aminosalicylic acid (5-ASA) against colitis, researchers designed a colon-specific mutual 5-ASA prodrug. Based on the beneficial effects of local anesthetics on colitis, procainamide (PA), pharmacologically classified as an antiarrhythmic and a local anesthetic [8], was linked to 5-aminosalicylic acid via an azo bond to produce PA-conjugated 5-ASA (5-ASA-azoPA, **1**) (Figure 3). Compound **1** released 5-ASA and PA in the large intestine after microbial azo-reduction to amines [9], presumably cooperating to ameliorate inflammation.

PA was selected because it is less lipophilic than other local anesthetics and has the potential to interfere with the activity of NFκB, an anticolitic drug target. The authors demonstrated that the therapeutic effects of **1** likely resulted from an additive anticolitic effect of 5-ASA and PA. The in vitro and in vivo data suggested that **1** could deliver 5-ASA and PA to the large intestine. As the conversion of the prodrug to 5-ASA and PA did not take place in the autoclaved cecal contents where microbial enzymes were inactivated, Kim et al. believed that the activation of the prodrug occurred by the microbial enzymes azoreductases. Oral gavage of **1** did not make PA detectable in the blood, while a substantial amount was detected after PA administration, suggesting that colonic delivery of PA can limit its systemic absorption. The combined intracolonic treatment with 5-ASA and PA achieved better anticolitic effects than treatment with 5-ASA alone. These data suggested that PA contributed to the anticolitic effects of **1**. Kim et al. showed that the combined treatment of 5-ASA-azo and PA reduced the levels of NFkB produced in the inflamed colon. However, from the data obtained, the significant therapeutic superiority of **1** to sulfasalazine, used as a reference compound, was not observed, especially in improving inflammatory indices such as CDS (colonic damage score), MPO (myeloperoxidase), and inflammatory mediators. Although it is not as active as sulfasalazine, it could have several advantages as it contains the local anesthetic procainamide. The reduction of abdominal pain and the improvement of the large intestinal functions by down-regulating overactive sympathetic nerves could be due to the conjugation with procainamide. Moreover, the side effects of sulfasalazine deriving from the sulfapyridine carrier, would thus be greatly reduced. After this study, Kim et al. discovered the potential colon-specific mutual prodrug activity of **1** against colitis, and this prodrug could act by cooperative NFκB inhibition [7].

### 3.2. Antidiabetic Properties

**Scheme 2:**
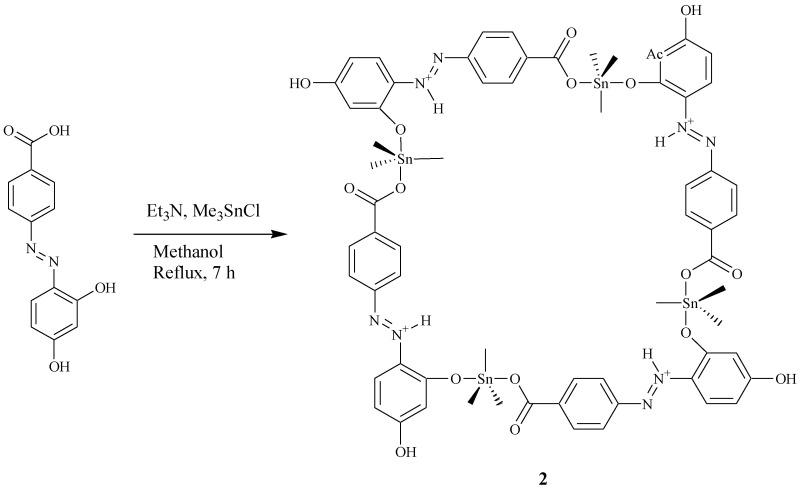
Synthesis of macro-cyclic tetrameric trimethyltin(IV) complex **2**.

M. Roy et al. synthesized triorganotin(IV) complexes of azo-carboxylic acids derived from amino benzoic acids and resorcinol. They were obtained by the reaction of 2/4-(2,4-dihydroxy-phenylazo)-benzoic acids with appropriate triorganotin(IV) chlorides in the presence of triethylamine (Scheme 2) [10]. From all synthesized phenylazo derivatives, the structure of compound **2** was established by X-ray crystal structure analysis, which revealed that this compound exhibited a 48-membered macrocyclic–tetrameric structure with trigonal bipyramidal geometry around the tin atoms, in which the three methyl groups occupied the equatorial positions, while the apical positions were occupied by the oxygen atom of the carboxylate group of one ligand and the phenoxide oxygen atom of another ligand.

Anti-diabetic activities of all synthetic complexes were studied using α-glucosidase enzymes and the results showed that some of them had significant results through the inhibition of more than 50% of α-glucosidase activity [10]. For example, complex **2** showed an IC_50_ value of 11.19 μg/mL compared to standard compound acarbose with an IC_50_ value of 12.15 μg/mL. Since the compounds exhibited more effective anti-diabetic activity than the standard compound, this promising result will also open a new avenue for researchers to investigate organotin(IV) azocarboxylates for their anti-diabetic activities.

### 3.3. Anti-Inflammatory Activities

**Scheme 3:**
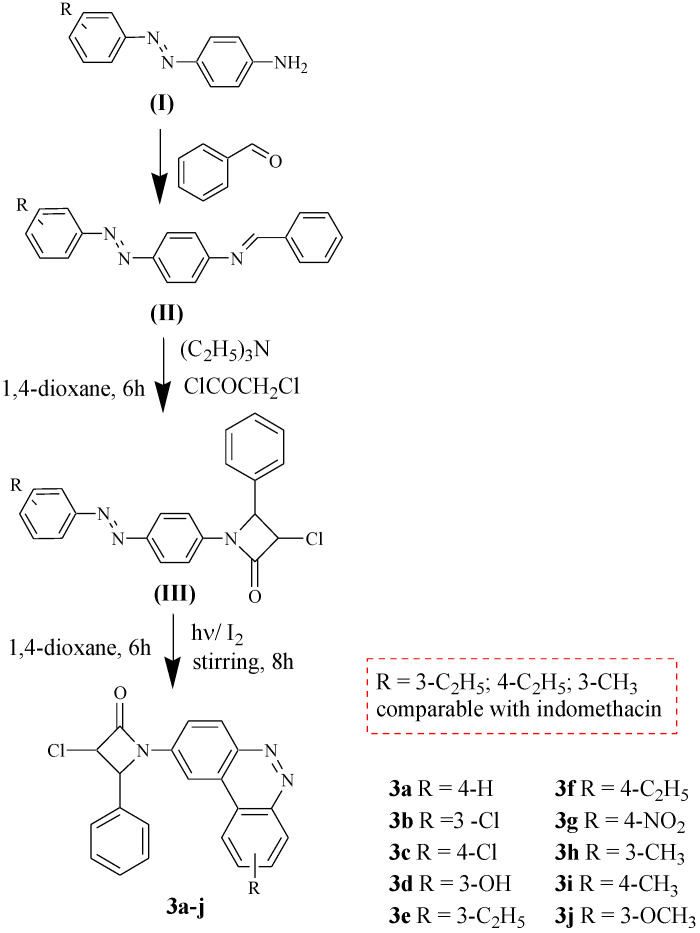
Overview of synthetic scheme for 3-chloro-1-(4a,10b-diazaphenanthrene-2-yl)-4-phenyl azetidin-2-ones (**3a**–**j**).

Inflammation is the body’s defense mechanism to protect against infection, burns, toxic chemicals, allergens, or other noxious stimuli. Sharma et al. reported a novel strategy to synthesize some 3-chloro-1-(4a,10b-diazaphenanthrene-2-yl)-4-phenyl azetidin-2-one derivatives (**3a**–**j**, Scheme 3) and explored their anti-inflammatory potential using rat carrageenan-induced paw edema tests [11]. The consequences of this mechanism can be manifold and they can induce, maintain, or aggravate many diseases. Using the reaction between *N*-{4-[phenyldiazenyl]phenyl}-*N*-[phenyl methylene]amine and 4-[phenyldiazenyl]aniline, new derivatives of 3-chloro-1-(4a,10b-diazaphenanthrene-2-yl)-4-phenylazetidin-2-one were synthesized (Scheme 3). The resulting 3-chloro-4-phenyl-1-{4-[phenyldiazenyl] phenyl}azetidin-2-one intermediate(III) in benzene was used to obtain the desired derivatives (**3a**–**j**) by irradiation in a Pyrex vessel with 350 nm UV light in a photochemical reactor. Structures of the new compounds were verified based on spectral and elemental methods of analyses. Compounds **3a**–**j** were tested for their anti-inflammatory effects using indomethacin as a reference compound. Compounds **3e**, **3f**, and **3h** exhibited almost comparable results to those of indomethacin 5 h after injecting them. Indeed, the percentages of the inhibitory effects of these three compounds upon carrageenan-induced paw edema in rats given indomethacin after 5 h were 90.80% for compound **3e**, 90.50% for **3f**, for 91.50% **3h**, and 92.25% for indomethacin [11].

### 3.4. Antimicrobial and Antimycotic Activities

**Figure 4:**
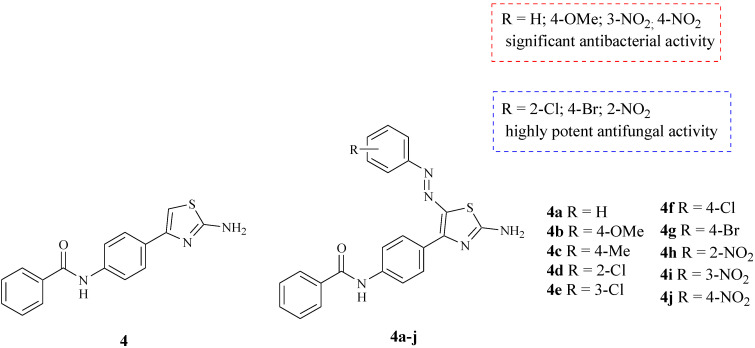
Chemical structures of phenylbenzamido-aminothiazole **4** and its derivatives **4a**–**j**.

Nowadays, bacterial and mycotic infections are the most important health problems. New antimicrobial agents that fight against these infections are necessary. Yadlapalli et al. synthesized a series of aminothiazole-ligated azo derivatives containing benzamide moiety (**4a**–**j**) as novel antibacterial and antifungal agents (Figure 4) [12].

The azoderivatives **4a**–**j** were tested to explore the antimicrobial activity by evaluating the Minimal Inhibitory Concentration (MIC) against *Escherichia coli*, *Enterobacter cloacae*, *Bacillus licheniformis*, and *Mycobacterium tuberculosis* (MTB) H37Rv as well as antifungal activity against three test phytopathogenic fungi.

The MIC values of the synthesized compounds were generally within the range of 7.8 × 10^−3^–2.5 × 10^−1^ mg/mL against all tested microorganisms. In particular, compound **4b** (R = 4-OMe) showed excellent activity (7.8 × 10^−3^ mg/mL) and compounds **4a**, **4i**, and **4j** exhibited good activity (6.25 × 10^−2^ mg/mL) against *Escherichia coli*. Compounds **4i** and **4j** also showed very good activity against the Gram-positive bacteria *Bacillus licheniformis* (6.25 × 10^−2^ mg/mL and 3.125 × 10^−2^ mg/mL, respectively). Out of all azo compounds, **4j** (R = 4-NO_2_) was found to exhibit significant activity against all three tested bacteria and showed activity of 1.25 × 10^−1^ mg/mL against *Enterobacter cloacae*. Compounds **4**, **4d**, **4g**, and **4h** were also found to be highly potent antifungal agents. In particular, compounds **4**, **4d**, **4g**, and **4h** showed 131, 180, 111, and 171 percent inhibition, respectively, better than the reference standard nystatin (100%), against *A. alternata*. Compounds **4** and **4d** showed 106 and 173 percent inhibition, respectively, better than nystatin (100%), against *C. lunata*. Compounds **4d**, **4g**, and **4h** (R = 2-Cl, 4-Br, and 2-NO_2_, respectively) showed 117, 207, and 149 percent inhibition, respectively, better than the standard, against *F. oxysporum* [12].

**Scheme 4:**
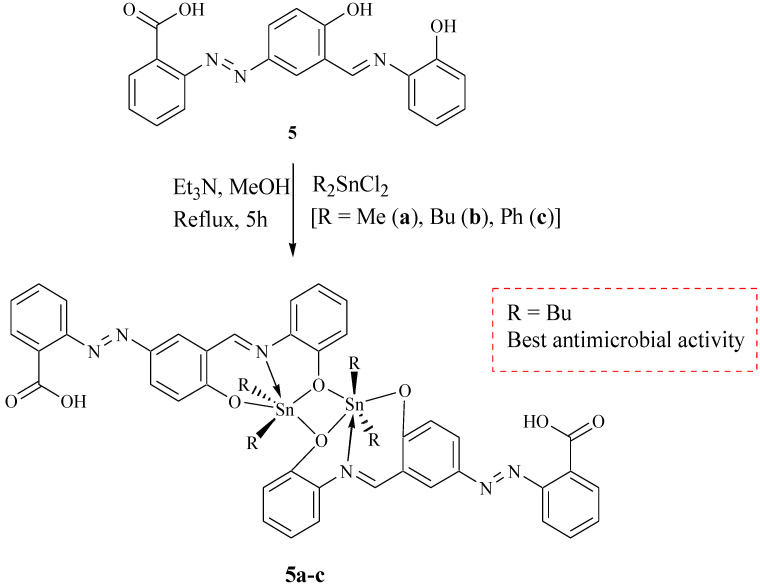
Scheme reaction for the synthesis of phenylazo diorganotin(IV) complexes **5a**–**c**.

Roy et al. synthesized some phenylazo diorganotin(IV) complexes (**5a**–**c**) according to Scheme 4 [13]**.**

These diorganotin(IV) complexes were evaluated for their antibacterial and antifungal activity. In particular, dibutyltin(IV) complex **5b** exhibited a lower MIC value at 4.2 μg/mL than the standard antibiotic gentamycin, which showed an MIC at 5 μg/mL against the bacteria *Staphylococcus aureus*, *Bacillus subtilis*, and *Bacillus cereus*. This complex also showed lower MIC values (8.5 μg/mL) than the standard compound nystatin, which exhibited an MIC at 10 μg/mL against *Candida albicans* and could be further studied to improve these antimicrobial therapeutic properties.

**Figure 5:**
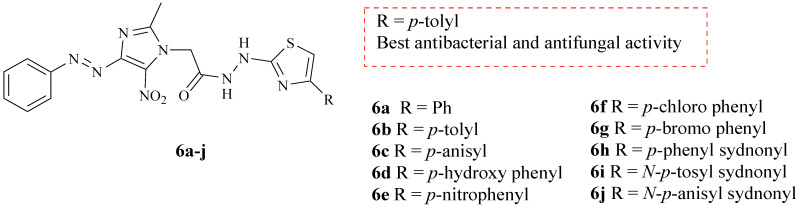
Chemical structures of (2-methyl-5-nitro-4-phenylazo–imidazol-1-yl)-acetic-acid-*N*-(4-phenyl-thiazol-2-yl)-hydrazide **6a**–**j**.

The potential antibacterial properties of azobenzene derivatives were also studied by Sreedevi et al., who synthesized azobenzene molecules containing thiazole and imidazole moieties (**6a**–**j**) and screened them for antimicrobial properties (Figure 5) against *Escherichia coli*, *Pseudomonas aeruginosa*, *Klebsiella pneumonia*, and *Staphylococcus aureus* [14].

Table 1 reports the antibacterial and antifungal activities of the most active tested compounds. Among these compounds, the *p*-tolyl derivative (**6b**) showed more antibacterial activity than that of the standard ciprofloxacin. *p*-Hydroxyphenyl (**6d**) and *N*-*p*-anisylsydnonyl (**6j**) showed considerable antibacterial activity. Antifungal activity studies carried out by the cup–plate method against *Candida albicans*, *Aspergillus flavus*, *Aspergillus fumigates*, *Trichophyton rubrum*, and *p*-tolyl (**6b**) showed higher activity than that of the standard ciclopiroxolamine, while *p*-hydroxyphenyl (**6d**) and *N*-*p*-anisylsydnonyl (**6j**) showed considerable antifungal activity.

**Figure 6:**
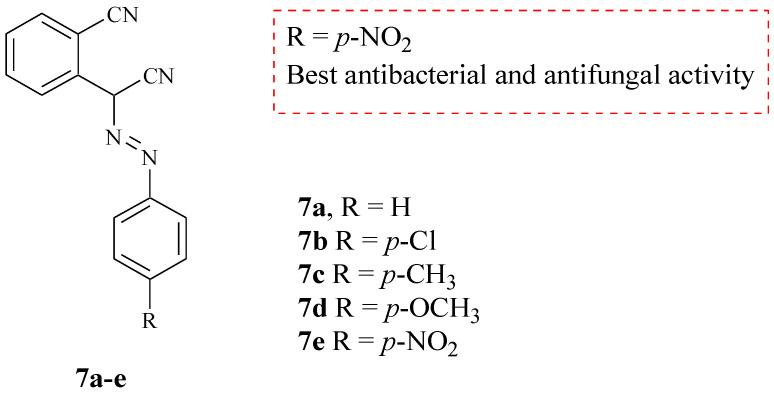
Chemical structures of (*E*)-2-(cyano(aryldiazenyl)methyl)benzonitrile derivatives **7a**–**e**.

Some aryldiazenyl derivatives were synthesized by Fadda et al. with the aim of investigating their antibacterial activity against Gram-positive bacteria, Gram-negative bacteria, and antifungal activity (Figure 6) [15]. All synthesized compounds were evaluated for their in vitro antibacterial activity against *Bacillus subtilis* and *Bacillus thuringiensis* as Gram-positive bacteria and *Escherichia coli* and *Pseudomonas aeruginosa* as Gram-negative bacteria. They were also evaluated for their in vitro antifungal potential against the *Fusarium oxysporum* and *Botrytis fabae* fungal strains. In particular, against Gram-positive bacteria, compounds **7a**, **7b**, **7d**, and **7e** exhibited broad-spectrum antibacterial profiles. Compounds with electron-withdrawing groups such as *p*-NO_2_ (**7e**) and Cl (**7b**) recorded higher activity. In this view, these compounds showed higher activity in the order of **7e** > **7b** > **7a** > **7d**. Also, compounds **7e** and **7b** were equipotent to chloramphenicol and ampicillin in inhibiting the growth of *B. subtilis* (MIC 3.125 mg/mL), while their activity was 50% lower than that of chloramphenicol against *B. thuringiensis*. Among all compounds, (*E*)-2-(cyano((4-nitrophenyl)diazenyl)methyl)benzonitrile (**7e**) (Figure 6) exhibited significant activity toward both Gram-positive and Gram-negative bacteria and exhibited the most potent in vitro antifungal activity, with an MICs of 6.25 μg/mL against *Botrytis fabae*.

**Figure 7:**
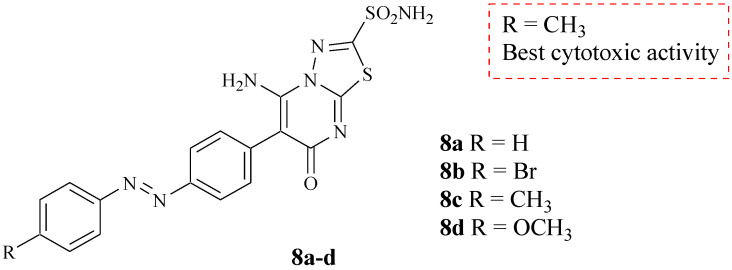
Chemical structures of compounds **8a**–**d**.

Antimicrobial resistance and the rapid rate of microbial evolution have led researchers to develop novel antibiotics. For example, El-Gohary et al. synthesized different azobenzene derivatives (Figure 7) that were screened for in vitro antimicrobial activity against two species of Gram-positive bacteria, *Staphylococcus aureus* and *Bacillus cereus,* and one species of Gram-negative bacteria, *Escherichia coli* [16]. Antifungal assays against *Candida albicans*, *Aspergillus fumigatus*, and *Aspergillus flavus* were also performed. The phenyldiazenyl moiety increased the activity against *Staphylococcus aureus* and, in particular, the thiadiazolopyrimidine nucleus possessing the methyl substituent at the *para* position of the 6-phenyldiazenyl moiety (**8c**) increased the antifungal activity against *Candida albicans*. Moreover, compound **8c** was also screened for cytotoxic activity against brine shrimp larvae (nauplii) and showed good cytotoxic activity in comparison with 5-flurouracil, with a lethal concentration (LC_50_) value of 392.46 μg/mL.

**Figure 8:**
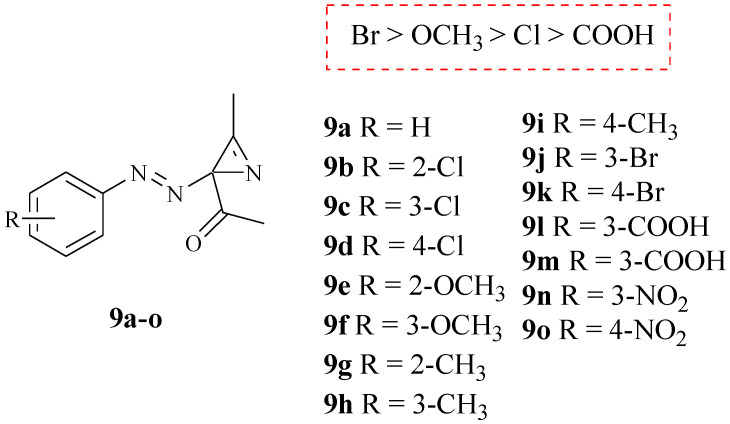
Chemical structures of compound 1-[3-methyl-2-(aryldiazenyl)-2*H*-aziren-2-yl]ethanone derivatives **9a**–**o**.

Other important results were obtained by Sahu et al., who synthesized a series of 1-[3-methyl-2-(aryldiazenyl)-2*H*-aziren-2-yl]ethanones compounds that exhibited good antimicrobial activity (Figure 8) [17]. All compounds were tested against *Bacillus subtilis*, *Escherichia coli*, *Pseudomonas diminuta*, and *Staphylococcus aureus* using chloramphenicol as a reference drug and exhibited remarkable in vitro activity within an MIC range of 2–57 μg/mL. The antimicrobial activity followed the pattern *B. subtilis* > *S. aureus > P. diminuta > E. coli.*

In particular, the compound with the bromo substituent, **9j**, was found to be the most potent among this series, with 4.441, 4.429, 4.457, and 4.425 -log MIC against *B. subtilis, S. aureus, P. diminuta,* and *E. coli*, respectively. The in vitro antimicrobial profile of 1-[3-methyl-2-(aryldiazenyl)-2*H*-aziren-2-yl]ethenone (**9a**–**o**) increased when substituents such as –Br, –OCH_3_, –Cl, and –COOH were present at the aromatic ring of azirine analogs. Moreover, it is further evident that *meta* –Br, –OCH_3_ and *ortho* –Cl, and –COOH analogs exhibit high biological potential.

**Figure 9:**
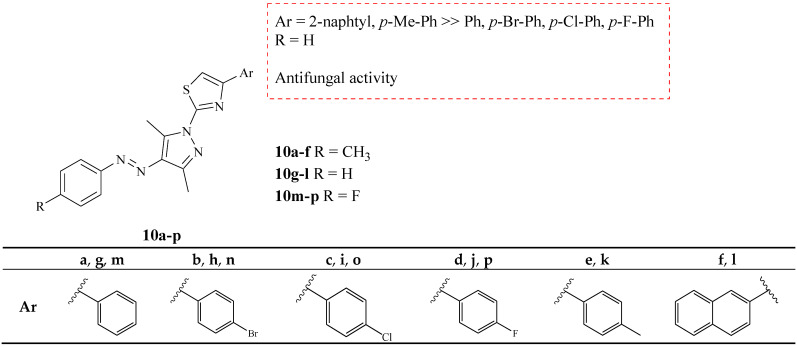
Chemical structures of (*E*)-2-(3,5-dimethyl-4-(aryldiazenyl)-1*H*-pyrazol-1-yl)-4-arylthiazole compounds **10a**–**p**.

In search of novel antibacterial compounds, Kaur et al. synthesized some aryldiazenyl azole derivatives (Figure 9) [18].

To evaluate the antimicrobial potential, compounds **10a**–**p** were screened for their in vitro antibacterial and antifungal activity using ciprofloxacin and amphotericin-B as positive controls for bacteria and yeasts, respectively. From this study, it was found that all compounds (except **10c** and **10e**) were found to be active against the yeast strain of *C. albicans* (inhibition zone (IZ) in the diameter range = 12–50 mm). Among them, compound **10l** showed a very big inhibitory zone of diameter 50 mm, while **10g**–**k** displayed zones of 21–25 mm in reference to amphotericin-B (IZ = 16.6 mm). Three compounds, **10a**, **10b**, and **10d**, were found to be active against *E. coli* (IZ = 12 mm), and none of the compounds possessed activity against *B. subtilis*, *S. aureus*, *P. aeruginosa*, or *S. cerevisiae*. Also, MICs were measured in μg/100 μL, and it was found that **10l** exhibited two times the inhibitory potential (MIC = 6.25) against *C. albicans* in comparison with the standard drug (MIC = 12.5), while compound **10k** (MIC = 12.5) was found to be equipotent. On the other hand, compounds **10g**–**j** exhibited two-fold lesser inhibitory action, with an MIC = 25 against *C. albicans* compared to the reference drug. Compounds with aryl groups at position 4 of the thiazole nucleus in the presence of R = H to position 4 of the pyrazole ring emerged as potent antifungal agents that possessed very high inhibitory action selectively against *Candida albicans*, a yeast strain. Compound **10l** was the most active agent, even more so than the reference antifungal drug. Compound **10k** bearing R = H and Ar = *p*-methylphenyl exhibited an inhibitory potency similar to the standard drug against *Candida albicans*. Therefore, compounds **10l** and **10k** could serve as new antifungal agents.

**Figure 10:**
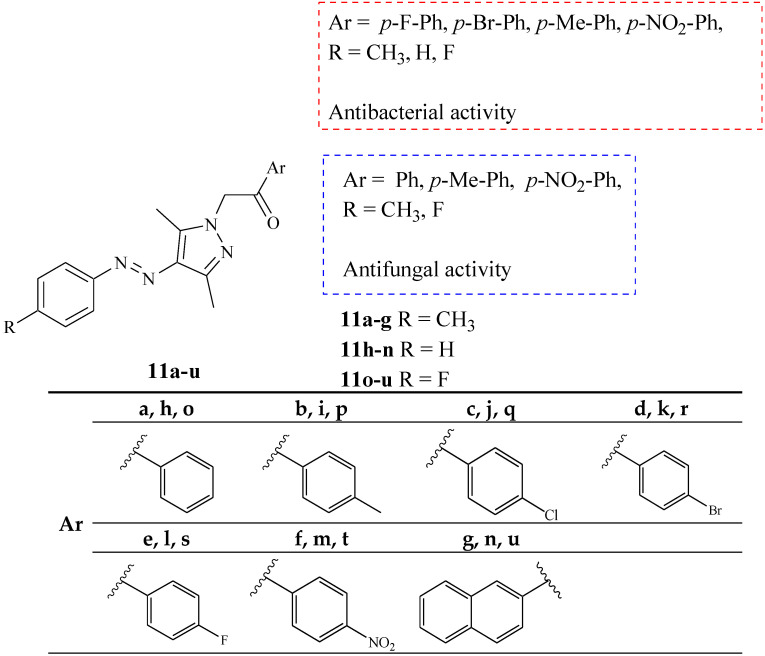
Chemical structures of (*E*)-1-aryl-2-(3,5-dimethyl-4-(aryldiazenyl)-1*H*-pyrazol-1-yl)ethanones compounds **11a**–**u**.

The same authors published another work in which the synthesis of a similar series of aryldiazenyl pyrazol derivatives **11a**–**u** (Figure 10) with antibacterial and antifungal activity was described [19]. In antibacterial screening (on the basis of MIC values), it was found that compounds **11b** and **11m** exhibited high inhibitory potential against *P. aeruginosa*, with an MIC = 25, which was about 50% that of ciprofloxacin (MIC = 12.5). The antifungal evaluation (on the basis of MIC values) revealed that compounds **11g**, **11o**, and **11p** exhibited two-fold lesser inhibitory potential selectively against *C. albicans* (MIC = 25). However, compound **11t** displayed two-fold lesser action against *S. cerevisiae* and four-fold lesser inhibitory potential against *C. albicans* (MIC = 50). On the other hand, **11a**–**c**, **11e**, **11i**–**n**, **11r**, and **11u** exhibited moderate inhibitory profiles against two fungal strains, with s = 50, about 25% of amphotericin B. Compounds **11d**, **11h**, **11q**, and **11s** exhibited four-fold lesser inhibition against *C. albicans* (MIC = 50) only. Among the series, compounds **11b**, **11i**, **11k**–**m**, and **11t** were found to be good antibacterial agents; however, the four compounds **11g**, **11o**, **11p**, and **11t** emerged as an excellent class of antifungal agents.

**Figure 11:**
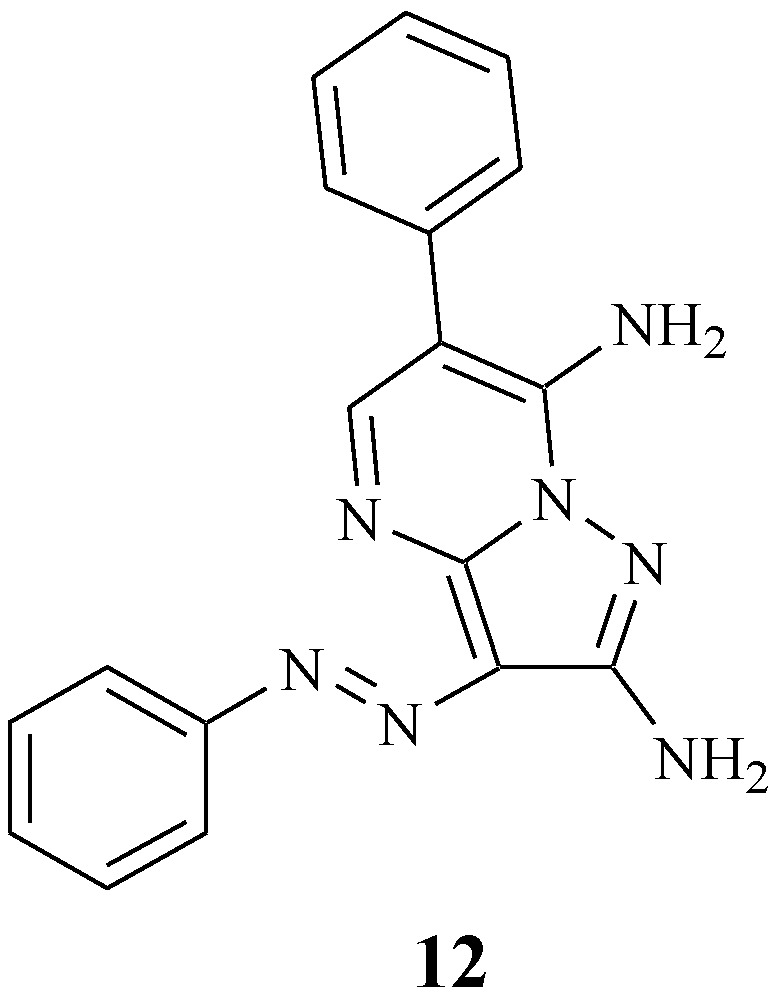
Chemical structure of 2,7-diamino-6-phenyl-3-phenylazopyrazolo [1,5-a]pyrimidine (**12**) compound.

Recently, Aggarwal et al. reported a phenylazo derivative as a new heterocyclic ligand that acts as a selective Hg^2+^ ion chemosensor and antimicrobial agent (Figure 11) [20].

This compound was tested on seven different clinical bacterial strains, namely, *Pseudomonas aeruginosa*, *Escherichia coli*, *Salmonella typhi*, *Staphylococcus aureus*, *Listeria monocytogenes*, *Bacillus cereus*, *Shigella flexneri*, and *Candida albicans*, as a fungal strain and displayed high antibacterial activity with the inhibition of all experimental microbial strains used at 3.125 μg/mL MIC.

**Figure 12:**
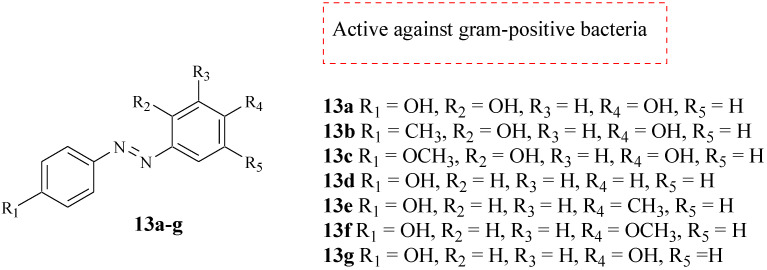
Chemical structures of 4,4′-dihydroxy-azobenzenes **13a**–**g**.

The increase in antibiotic resistance is one of the most significant public health concerns. For this reason, there is increasing interest in the synthesis of new antibiotic compounds. Recently, compound 4,4′-dihydroxy-azobenzene **13g** (Figure 12) was tested in vitro as an antimicrobial agent against *Staphylococcus aureus* and *Staphylococcus pseudintermedius* by Pérez-Aranda et al. [21]. This molecule is a derivative of other azo compounds (**13a**–**f**) active as antimicrobials reported in Figure 12 [22,23].

The values of the MICs of compound **13g** against *Staphylococcus aureus* and *Staphylococcus pseudintermedius* were 64 and 32 μg/L, respectively, and comparable to its azo parent antimicrobial compounds described in the literature [22]. Moreover, the minimal bactericidal concentrations (MCBs) were 256 and 64 μg/L, respectively. Furthermore, **13g** was also tested against *Escherichia coli* and *Pseudomonas aeruginosa*, but it did not show good results. These data are in accordance with those of azo compounds **13a**–**f**, indicating that these substances can effectively inhibit the growth of Gram-positive bacteria, but they are not useful for Gram-negative bacteria.

**Figure 13:**
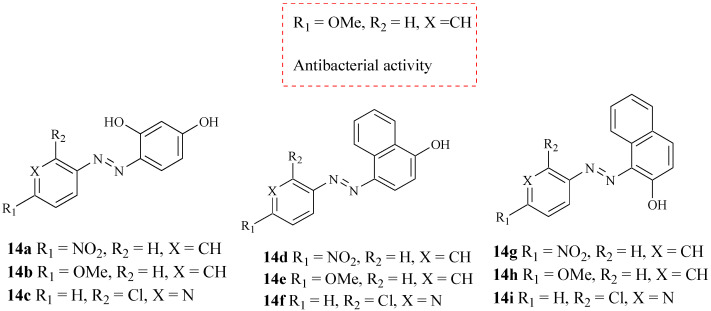
Chemical structures of azo derivatives **14a**–**i**.

In another study, Tahir et al. synthesized diaryl azo-phenol derivatives **14a**–**i** (Figure 13) because of the synthetic feasibility of azo derivatives and the multiple therapeutic properties of phenolic compounds fused with azo moiety [24].

All these azo compounds were tested as antimicrobials against pathogenic bacterial strains *Escherichia coli* (Gram-negative), *Staphylococcus aureus* (Gram-positive), *Staphylococcus aureus* (drug-resistant strain), *Pseudomonas aeruginosa* (Gram-negative), *Pseudomonas aeruginosa* (drug-resistant strain), and *Proteus vulgaris* (Gram-negative). Compounds **14a**, **14b**, and **14e** displayed promising antibacterial potential, with an average MIC value of 125 μg/200 mL, and were found to be dose-dependent compared to the standard drugs, i.e., ampicillin and moxifloxacin (MIC 62.5 μg/200 mL). These series were also evaluated against alpha-glucosidase (anti-diabetic), and the IC_50_ of **14a** was found to be most effective (15.70 ± 1.3 μ/mL) compared to the reference drug acarbose (21.59 ± 1.5 μ/mL); this compound can be considered a potential multi-target compound.

**Figure 14:**
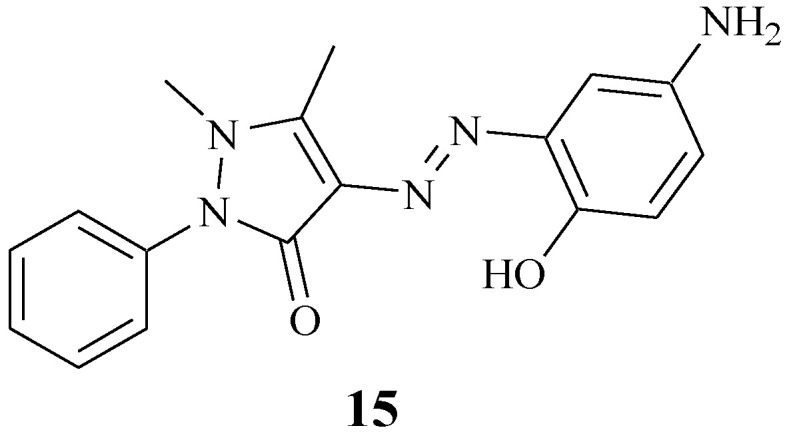
Chemical structure of the azo derivative **15**.

Recently, Al-Gaber et al. synthesized the new heterocyclic azo dye ligand **15** by a combination of 4-amino antipyrine and 4-aminophenol (Figure 14) [25]. Moreover, the coordination of this phenylazo compound was investigated with metal ions such as Cr(III), Mn(II), Fe(III), Co(II), Ni(II), Cu(II), Zn(II), and Cd(II), and the antimicrobial properties of the azo dye ligand and its metal chelates were also assessed using ketoconazole and amikacin as the standard antifungal and antibacterial agents, respectively. Most complexes were more potent than the free ligand against fungi and bacteria. For example, the Fe(III) metal chelate was the most effective against *Aspergillus fumigatus* and *Escherichia coli.* The azo dye ligands and Mn(II), Co(II), Ni(II), and Zn(II) metal chelates showed the highest antibacterial potential against *S. aureus* and the Zn(II), Ni(II), and Cr(III) complexes showed the highest antifungal activity against *Candida albicans*. Besides that, the Co(II) and Cr(III) complexes showed the most promising antibacterial activity against *Salmonella sp* bacteria and were higher than the antifungal standard. In contrast, the Cu(II) metal chelate showed the highest antibacterial activity against the *Bacillus subtilis* bacteria. This study highlighted that the antimicrobial action of azo dye compounds may be significantly enhanced by the presence of an azo group with chelating properties.

**Figure 15:**
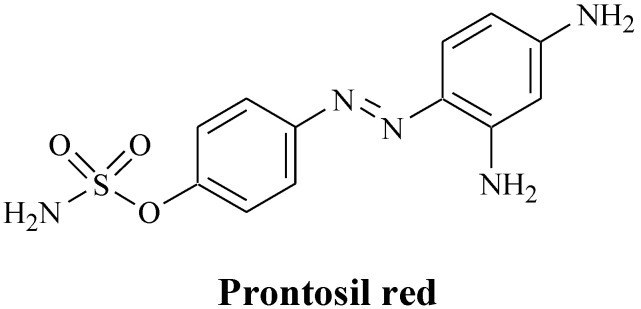
Chemical structure of prontosil red.

Prontosil red (Figure 15) was the first antibacterial azo dye to be discovered in 1932 from Bayer’s laboratory [26].

The importance of the sulfonamide group is crucial; several studies demonstrated that this moiety is responsible for the therapeutic effect of the compound [27,28]. This led researchers to synthesize and test novel azo compounds containing the sulfa group, obtaining sulfonamides and sulfamates with the aim of evaluating the antimicrobial and antiviral activities. These two moieties are in a diverse family of highly pharmacologically active compounds that contain the signature sulfamoyl structural motif.

**Figure 16:**
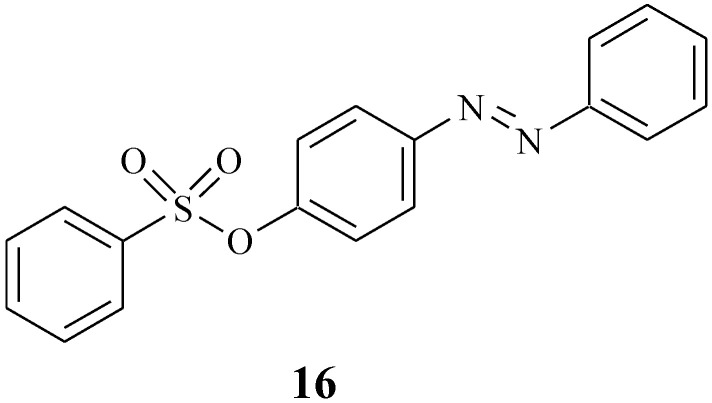
Chemical structure of 4-(phenyldiazenyl)phenyl benzene sulfonate **16**.

For example, Moanta et al. synthesized 4-(phenyldiazenyl)phenyl benzene sulfonate **16** (Figure 16), which was screened for its antimicrobial activity against two Gram-positive bacteria (*Staphylococcus aureus* and *Streptococcus pyogenes*), three Gram-negative bacteria (*Pseudomonas aeruginosa*, *Proteus vulgaris*, and *Escherichia coli*), and two fungi species (*Candida albicans* and *Aspergillus niger*) [29]. The maximum relative percentage of inhibition was exhibited against *Candida albicans* (85.20%), followed by *Staphylococcus aureus* (83.99%), *Aspergillus niger* (66.94%), *Escherichia coli* (42.94%), *Pseudomonas aeruginosa* (38%), *Proteus vulgaris* (17.07%), and *Streptococcus pyogenes* (13.30%), respectively.

### 3.5. Anticancer Activities

**Figure 17:**
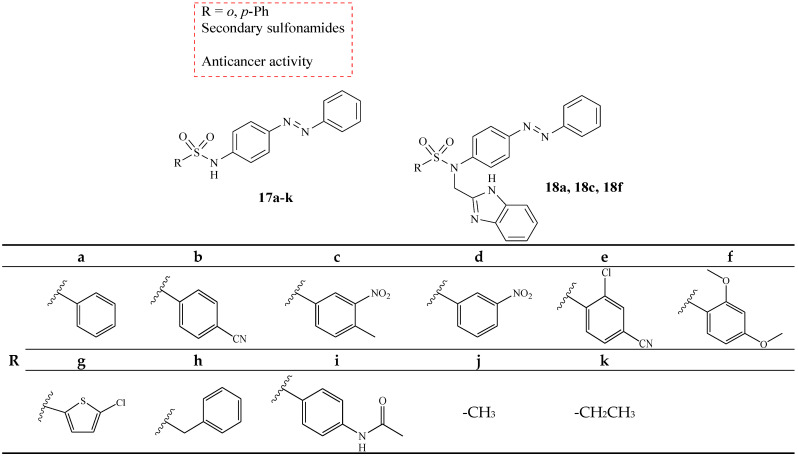
Chemical structures of compounds **17a**–**k** and **18a**, **18c**, and **18f**.

Several in vitro and in vivo studies have confirmed that azo compounds could potentially act as anticancer agents. Recently, Giampietro et al. reported the synthesis, biological evaluation, and docking studies of a series of phenyldiazenyl sulfonamide compounds as aromatase inhibitors (AIs) (Figure 17) [30]. In these molecules, the classical bioisosterism in the stilbenoid nucleus, which also showed aromatase inhibition [31], was used to replace the C=C bond present in the bridge between the two phenyl rings of stilbene with the isosteric N=N bond, obtaining azostilbene derivatives.

Three compounds, **17a**, **17f**, and **18f**, showed a percentage of enzymatic inhibition better than letrozole, used as a reference compound, and IC_50_ values in the micromolar range (7.9, 1.6, and 5.7 μM, respectively). These three compounds were also evaluated in vitro on MCF-7 breast cancer cells by MTT and cytotoxicity assays. The best results were obtained for compound **17f** and confirmed by the analysis of cell cycles and apoptosis. A significant dose-dependent increase in the percentage of cells found in the apoptotic stage was assessed in the presence of **17f**; moreover, **17f** showed an anti-proliferative effect on MCF-7 cells, being blocked in the G1/S phase checkpoint.

Furthermore, **17f** was also not effective on HFF-1, showing an increase in the selectivity index from 24 h to 72 h. Computational studies were carried out and gave a sound explanation at the molecular level of the experimental data. Compound **17f** can be considered an interesting lead for the development of a new class of non-steroidal aromatase inhibitors.

**Figure 18:**
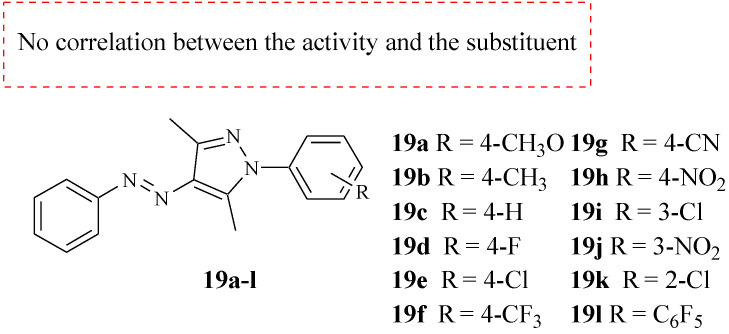
Chemical structures of compounds **19a**–**l**.

In research on new anticancer compounds, Bustos et al. synthesized a series of twelve phenyldiazenyl pyrazoles (**19a**–**l**) and tested these compounds against cancer cell lines over a wide library of cell lines, including leukemia, colon, and brain cancer, showing that the compounds have anticancer activity (Figure 18) [32].

All compounds showed a certain degree of growth inhibition against different cell lines. Furthermore, all compounds also showed some degree of lethality against different cell lines, without a correlation between the activity and the substituent. However, it is important to note that highly selective growth inhibition activity was observed against the lung cancer cell line, kidney cancer cell line, and leukemia cell line.

**Figure 19:**
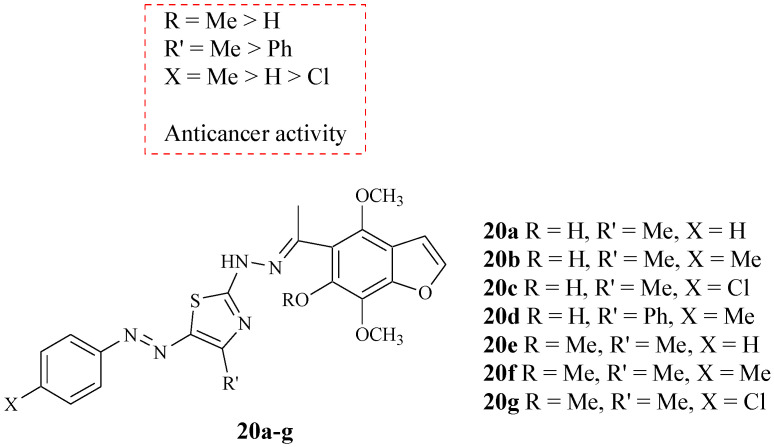
Chemical structures of compounds **20a**–**g**.

With the aim of identifying new anti-breast-cancer compounds, Gomha et al. synthesized a series of thiazole-benzofuran phenyldiazenyl compounds **20a**–**g** (Figure 19) [33]. These molecules were evaluated for their anticancer activity against the human breast carcinoma (MCF-7) cell lines compared with the doxorubicin drug (Table 2).

The descending order of activity of these compounds was as follows: **20f** > **20b** > **20e** > **20a > 20c** > **20d** > **20g**. In particular, it was possible to conclude that the anticancer activity depended on the structural skeleton and electronic environment of the molecules. Indeed, the introduction of an electron-donating group (methyl) at C4 of the phenyl group at position 5 in the 1,3-thiazole ring (**20f** and **20e**) enhanced the antitumor activity. In contrast, the introduction of an electron-withdrawing group (chlorine) decreased the antitumor activity (**20b** > **20a** > **20c**). Moreover, the in vitro inhibitory activity of the 6-methoxybenzofuran was higher than 6-hydroxybenzofuran (**20e** > **20a**, **20f** > **20b** and **20g** > **20c**), probably due to the +I (inductive) effect of the methyl group. The activity of 4-methylthiazole was greater than that of 4-phenylthiazole (**20b** > **20d**), also in this case because of the +I effect of the methyl group.

**Scheme 5:**
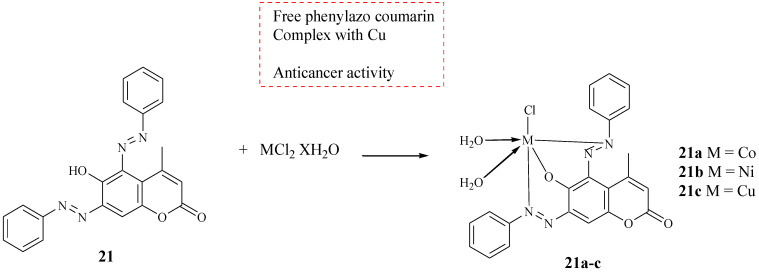
Synthesis of metal complexes **21a**–**c** starting from 6-hydroxy-4-methyl-5,7-(bisphenylazo) coumarin **21**.

In another study, Ibrahim et al. synthesized phenylazo coumarin **21** and its complexes with Co(II) (**21a**), Ni(II) (**21b**), and Cu(II) (**21c**) (Scheme 5); they evaluated the cytotoxic activity of the ligand and complexes against breast cancer cells [34].

The test revealed that cell proliferation was much more highly inhibited by the ligand **21** and complexes Cu (**21c**), Co (**21a**), and Ni (**21b**), with cell viability of 5.21%, 17.36%, 46.20%, and 74.43%, respectively, at a concentration of 30 mg/mL compared to untreated control cells, and the IC_50_ values of ligand **21** and complexes of Cu (**21c**), Co (**21a**), and Ni (**21b**) were 1.87, 1.87, 30, and >30 g/mL, respectively. This study demonstrated that ligand **21** and its complex with Cu (**21c**) had the most promising phenylazo coumarin activity against breast cancer cells.

Recently, Tang et al. synthesized different azobenzene coumarin derivatives [35]; between them, compounds **22a**–**I** (reported in Table 3), where the coumarin moiety was linked to substituted phenyl and pyridine groups via amide bonds, were tested to evaluate their anticancer activities. Compounds **22a**–**i** showed different degrees of anticancer activities against four cancer cell lines (HeLa, A549, MCF-7, and HepG-2). Among them, fluorobenzene-modified 6-azophenylcoumarin-3-formamido derivative **22f** exhibited stronger cytotoxicity (IC_50_ = 0.51 ± 0.22 μM) against A549 cell lines and was less toxic to normal cells than doxorubicin (DOX) (IC_50_ = 1.18 ± 0.03 μM). Pyridine-modified 6-azophenylcoumarin-3-formamido derivative **22g** exhibited stronger cytotoxicity against MCF-7 cell lines than DOX, with an IC_50_ value 48 times higher than that of DOX (IC_50_ = 0.42 ± 0.23 μM for **22g**; IC_50_ = 20.64 ± 3.67 μM for DOX). Meanwhile, compound **22g** was much less toxic to normal human umbilical vein endothelial cells (HUVECs) than DOX, with a 3000 times higher selectivity index (SI) (SI > 238.10 for **22g**; SI = 0.078 for DOX).

In particular, analyzing the data in Table 3, compounds **22e** and **22g** exhibited high biological activity against A549 and MCF-7 cancer cells, respectively. Moreover, compound **22g** not only exhibited strong cytotoxicity against MCF-7 cell lines but also was less toxic to normal cells, with SI > 238.10. These two compounds also induced apoptosis and arrested the cell cycle of A549 and MCF-7 cells in the S phase in a dose-dependent manner and were proven to bind to HS-DNA via intercalation.

### 3.6. Carbonic Anhydrase Inhibitors

Azo compounds have been studied for their potential carbonic anhydrase (CA) inhibition. For example, Arslan et al. reported a series of azo sulfonamide derivatives (**23a**–**f**) incorporating substituted chalcone moieties that are able to inhibit CA isoforms I and II [36].

The data (reported in Table 4) showed good results, and the selectivity of the inhibitors was distinguishable. The best performer against hCA I was compound **23f**, with a K_I_ of 9.88 nM. Other compounds (**23** and **23a**–**e**) showed powerful inhibitory profiles compared to acetazolamide (AZA), with Kı values between 13.25 and 24.4 nM. Against hCA II, all new compounds showed good results, with Kıs ranging between 18.25 and 55.43 nM. Compound **23a** (K_I_ 18.25 nM) is a promising CA I, with inhibition properties comparable to the clinically used sulfonamide, AZA.

**Figure 20:**
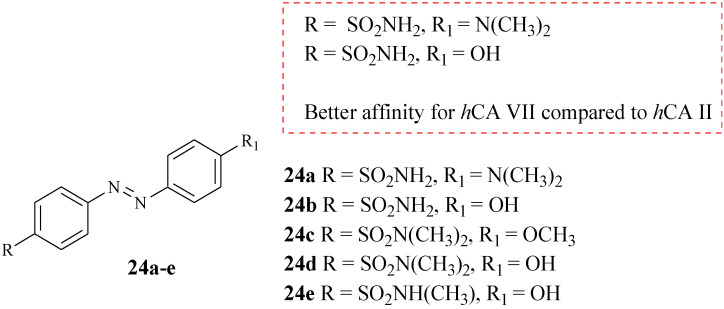
Chemical structures of azobenzenes **24a**–**e**.

*p*-phenyldiazenyl derivatives **24a**–**e** (Figure 20) were also synthesized and tested as CA inhibitors by Carta et al. [37].

Diazo dyes **24a**–**e** showed weak *h*CA II inhibitions, with inhibition constants (K_I_) in the range of 277–863 nM compared to that of AZA (12 nM). It is possible to observe that derivative **24c** with three methyls in its structure (K_I_ = 863 nM) was the worst *h*CA II inhibitor compared to its des-methylated congeners **24b**, **24d**, and **24e**, which showed K_I_ values of 665 nM, 312 nM, and 277 nM, respectively. Indeed, these compounds possessed either a SO_2_NH_2_, SO_2_NHMe, or OH moiety that could interact either with the Zn(II) ion or the zinc-bound water molecule, thus acting as better inhibitors than **24c**, in which only hydrophobic interactions between the inhibitor and the enzyme active site could occur. Azo dyes **24a** and **24b** revealed a better affinity for *h*CA VII compared to *h*CA II and referred to AZA (K_I_ = 2.5 nM). Thus, these *p*-phenyldiazenyl derivatives showed good selectivity for inhibiting *h*CA VII (K_Is_ of 30–46 nM against *h*CA VII) over *h*CA II (K_Is_ of 638–665 nM), as already mentioned.

The possibility of finding new *h*CA VII inhibitors could be interesting and help researchers better understand this cytosolic enzyme, largely expressed in the human brain, in order to develop new classes of compounds with better CA VII selective profiles.

**Figure 21:**
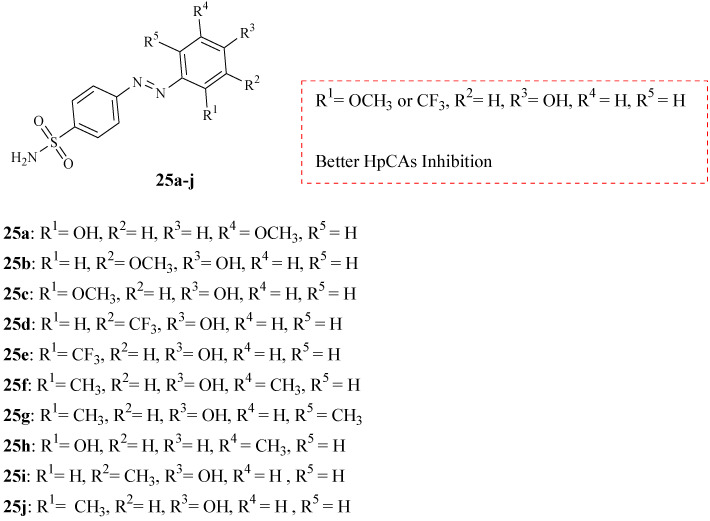
Chemical structures of azobenzenes **25a**–**j**.

Recently, a new series of azobenzenesulfonamides (Figure 21) were synthesized and tested against a large panel of human and bacterial CAs to evaluate their inhibitory activity [38]. In particular, compounds **25a**–**j** were tested versus *H. pylori* because targeting *H. pylori* Carbonic Anhydrases (HpCAs) represents a good option for the treatment of infections sustained by the pathogen’s drug-resistant strains [39]. Compounds **25c**,**e** reported in Figure 21 were the best, with interesting inhibitory activities on HpCAs.

For HpαCA, compound **25c** showed a Kis value of 70.9 nM, while the trifluoromethyl derivative **25e** showed a Kis value of 89.2 nM. KIs on *E. coli* CA from the β-class had low nanomolar concentrations, with the lowest values shown by **25c** and **25e** (KI = 17.1 and 68.8 nM, respectively). To further demonstrate the antimicrobial potential of these two compounds, they were also tested in vitro against the reference strain *H. pylori* ATCC 43504, and the Minimal Inhibitory Concentrations (MICs) and Minimal Bactericidal Concentrations (MBCs) were studied. Compounds **25c** and **25e** showed the same values for the MIC and MBC (4 and 16 μg/mL, respectively). Moreover, the evaluation of their toxicity on a *G. mellonella* larva in vivo model indicated a safe profile for **25c** and **25e**, considering these azobenzenesulfonamides as an interesting starting point for the development of a new class of anti-*H. pylori* agents.

### 3.7. COX-2 Inhibitors

**Figure 22:**
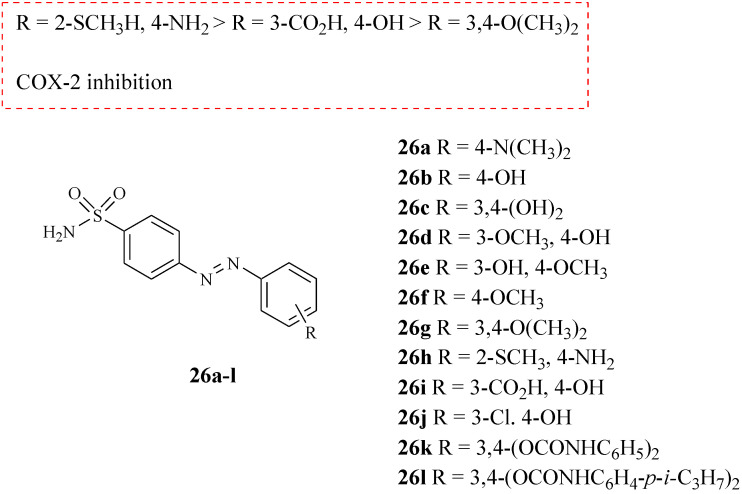
Chemical structures of phenylazobenzenesulfonamide derivatives **26a**–**l**.

A further field of application for azobenzene compounds is that of the inhibition of cyclooxygenase-2 (COX-2) enzymes, as Tsai et al. demonstrated in their study [40]. The researchers designed and synthesized phenylazobenzenesulfonamide derivatives **26a**–**l** (Figure 22) for their evaluation as selective COX-2 inhibitors in a cellular assay using human whole blood (HWB) and an enzymatic assay using purified ovine enzymes. To identify the selective inhibitor of COX-2, these series of compounds were subject to structure–activity relationship analyses, and several selective COX-2 inhibitors were identified. For example, compound 3,4-dihydroxyl (**26c**) exhibited moderate COX-2 inhibitory potency (IC_50_ = 12.42 μM). However, the replacement of the 3-hydroxy moiety with 3-chloro (**26j**) retained some COX-2 potency (IC_50_ = 11.34 μM), but was less active than that of the 3-carboxy group (**26i**) (IC_50_ = 8.89 μM). The introduction of the 3,4-dimethoxy moiety in **26g** (IC_50_ = 4.28 μM) provided excellent activity and selectivity compared to the parent compound **26c**. Compound **26g** was found to be a preferential COX-2 inhibitor (IC_50_ = 4.28 μM) and compound **26h** displayed a greater COX-2 inhibitory profile when compared to phenylazobenzenesulfonamide in this series (IC_50_ = 2.04 μM).

### 3.8. Anti MAO-B Activity

**Figure 23:**
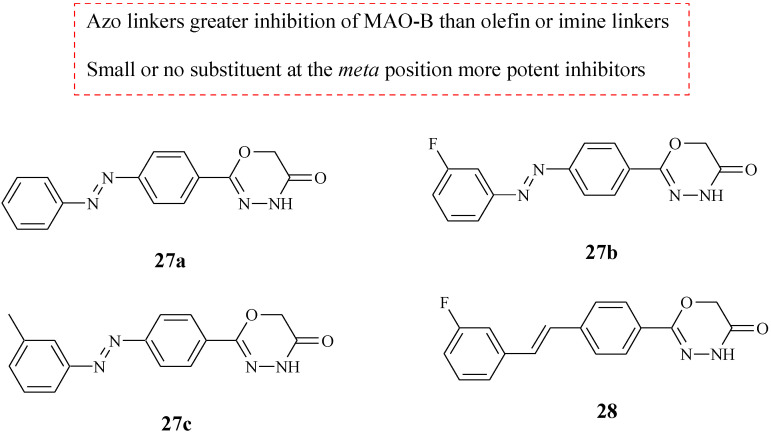
Chemical structures of compounds **27a**–**c** and **28** with the best activity against MAO-B.

One of the most common approaches to treating neurological disorders is the inhibition of monoamine oxidase B (MAO-B) [41]. MAO-B is a flavin adenine dinucleotide (FAD)-containing enzyme that plays a major role in the oxidative deamination of biogenic amines and neurotransmitters. Lee et al. reported a series of 2-aryl-1,3,4-oxadiazin-5(6*H*)-one derivatives based on an analysis of the binding sites of *h*MAO-A and *h*MAO-B [42]. They designed linear analogs of 2-aryl-1,3,4-oxadiazin-5(6*H*)-one with an additional phenyl ring (**27a**–**c**, **28**). In particular, these compounds (reported in Figure 23) potently inhibited MAO-B, with IC_50_ values of 4–25 nM and excellent SI over MAO-A (**27a** > 25,000, **27b** > 8333, **27c** > 4000, and **28** > 4545). According to docking results, which suggested that an optimal linker between two aromatic rings on the 2-aryl-1,3,4-oxadiazin-5(6*H*)-one scaffold was a key element in the binding and inhibition of MAO-B, compounds with azo linkers (**27a**–**c**) showed greater inhibition of MAO-B than compounds with olefin or imine linkers. In addition, compounds with a small (F, **27b**, and **28**) or no (**27a**) substituent at the *meta* position were more potent inhibitors in the presence of azo or olefin linkers, with IC_50_ values of 4, 12, and 22 nM, respectively. These results indicate that the steric effect of the substituent in the distal phenyl ring in the presence of an azo or an olefin linker plays a key role in the modulation of MAO-B inhibitory activity.

**Figure 24:**
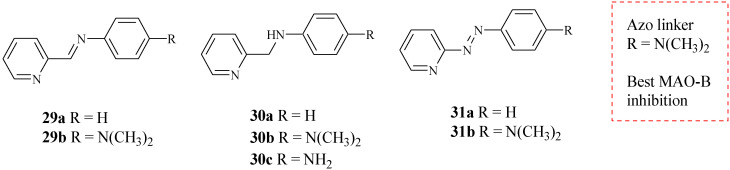
Chemical structures of compounds **29a**,**b**, **30a**–**c**, and **31a**,**b**.

Geldenhuys et al. focused their studies on the inhibition of MAO using a chemical library composed of stilbene-like derivatives that have potential applications in Alzheimer’s disease (AD) and dementia with Lewy bodies (DLB), with the ability to modulate metal-induced amyloid-β (Aβ) aggregation and neurotoxicity in vitro and in living cells [43]. From their studies, Geldenhuys et al. obtained some compounds (**29a**,**b**, **30a**–**c**, and **31a**,**b**) with potent and relatively selective inhibitory effects on MAO-B (Figure 24). In particular, azostilbene derivative **31b** showed a good inhibitory effect on MAO-B, with an IC_50_ of 0.14 μM. The dimethylamino moiety on these compounds is important for the MAO inhibition activity. These findings suggest that stilbene-like scaffolds and azo derivatives could be utilized to develop promising multifunctional, neuroprotective agents for several neurodegenerative diseases.

### 3.9. Neuroprotective Activities

**Figure 25:**
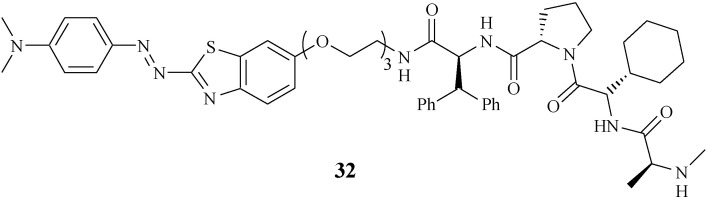
Chemical structure of azobenzene Htt degrader **32**.

Tomoshige et al. investigated the role of mutant huntingtin (mHtt), a protein whose aggregation caused the autosomal dominant neurodegenerative disorder Huntington’s disease (HD), assuming that protein knockdown could be a potential therapeutic option [44]. Inexpression of the protein is achieved by using hybrid small molecules (Htt degraders) consisting of BE04, a ligand of ubiquitin ligase (E3), linked to probes for protein aggregates. In their studies, Tomoshige et al. synthesized a similar Htt degrader utilizing MV1, an antagonist of the inhibitor of the apoptosis protein (IAP) family (a subgroup of ubiquitin E3 ligases), replacing the original ligand with one expected to have a higher affinity and specificity for IAP. A new Htt degrader **32** (Figure 25) was synthesized and showed a decrease in both wild-type Htt (wtHtt) and mHtt levels. Compound **32** induced an interaction between GST-BIR3 and insoluble model aggregates of 62Q peptides, supporting the idea that compound **32** works by forming a non-physiological complex consisting of Htt aggregate, IAPs, and compound **32**. In addition, this compound induced an interaction between GST-BIR3 and soluble GST-62Q, which is consistent with the finding that it reduces the levels of both mHtt and wtHtt.

**Figure 26:**
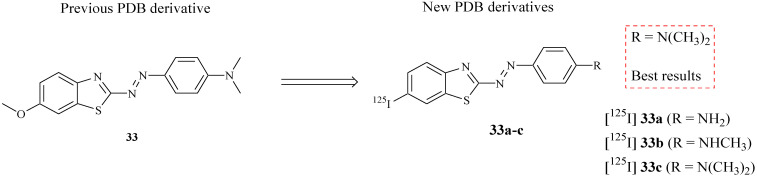
Chemical structures of compound **33** and PDB derivatives **33a**–**c**.

In the brain, senile plaques (SPs) composed of β-amyloid, peptides, and neurofibrillary tangles (NFTs) composed of hyperphosphorylated tau protein are considered the cause of AD, one of the most common neurodegenerative disorders. Based on previous studies in which the phenyldiazenyl benzothiazole (PDB) derivative 4-[2-(5-methoxy-2-benzothiazolyl)diazenyl]-*N*,*N*-dimethyl-benzenamine (**33**) (Figure 26) had a good ability to distinguish tau aggregates from Aβ aggregates [45], Matsumura et al. reported the synthesis and biological evaluation of novel PDB derivatives (**33a**–**c**) (Figure 26) as probes for imaging NFTs in patients with AD [46]. During the last few years, the use of radiolabeled probes has demonstrated promising applications in the field of nuclear medicine [47]. Based on this assumption, Matsumura et al. successfully synthesized these three PDB derivatives (**33a**–**c**) using a diazo coupling reaction [46]. From the in vitro obtained results, compound **33c** had more affinity with tau aggregates; also, from fluorescent staining experiments using AD brain sections, **33c** visualized NFTs clearly. From biodistribution assays with normal mice, the PDB derivatives showed uptake into the brain, sufficient for imaging NFTs, ranging from 0.94 to 3.2% ID g^−1^, but a relatively slow washout. This study could be useful to make new compounds with improved pharmacokinetics qualities obtained from some modifications.

### 3.10. Tyrosinase Inhibitors

Tyrosinase is a multifunctional enzyme that controls the production of melanin from tyrosine in animals [48]. The multifunctional, glycosylated, copper-containing metalloenzyme is involved in two distinct reactions of melanin synthesis: the hydroxylation of a monophenol and the conversion of an *O*-diphenol to the corresponding *O*-quinones. Based on previous studies that considered kojic acid, resveratrol, and azo-resveratrol as potential tyrosinase inhibitors, Bae et al. synthesized a novel series of (*E*)-2-((substituted phenyl)diazenyl)phenyl 4-methylbenzenesulfonate derivatives (**34a** and **34b**) and (*E*)-2-((substituted phenyl)diazenyl)phenol derivatives (**34c** and **34d**), conducting their evaluation on mushroom tyrosinase (Table 5) [49]. Compounds **34b**–**d** exhibited good inhibitory effects, higher than that of kojic acid (IC_50_ = 49.08 μM), a representative tyrosinase inhibitor. In particular, the novel synthesized compound (*E*)-2-((2,4-dihydroxyphenyl)diazenyl)phenyl 4-methylbenzenesulfonate (**34b**) was the best performer, with an IC_50_ of 17.85 μM. This molecule had competitive inhibition on Lineweaver–Burk plots, as further confirmed by the docking results. One of the most important goals of the study was the non-cytotoxicity of compounds **34b**–**d** to cultured B16F10 cells. These new derivatives inhibited both tyrosinase and melanin synthesis. For the development of new candidates against diseases associated with hyperpigmentation, active compounds (**34b**–**d**) might have an important role.

As previously reported, the natural compound resveratrol and its derivatives have potent antioxidant, anti-inflammation, anticancer, cardio-protection, and anti-tyrosinase activities [50,51]. Using a modified Curtius rearrangement and diazotization followed by coupling reactions with various phenolic analogs, Song et al. synthesized azo compounds (**35a**–**i**) (Table 6) including azo-resveratrol (**35b**) and azo-oxyresveratrol (**35f**) [52]. In the study, they replaced the linker between the two phenyl rings of resveratrol with an azo linker to obtain new non-symmetric azo compounds (**35a**–**i**). From all compounds evaluated for their mushroom tyrosinase inhibitory activity, compounds **35a** and **35b** exhibited high tyrosinase inhibitory activity (56.25% and 72.75% at 50 μM, respectively). Song et al. highlighted that the introduction of a hydroxyl or methoxy group into the 4-hydroxyphenyl moiety significantly reduced mushroom tyrosinase inhibition (Table 6). Azo-resveratrol (**35b**) was the most potent mushroom tyrosinase inhibitor, with a percentage of 72.75 ± 1.70 and an IC_50_ value of 36.28 ± 0.72 μM, comparable to that of resveratrol (26.63 ± 0.55 μM), a well-known tyrosinase inhibitor. The 4-hydroxyphenyl moiety is essential for high inhibition, and 3,5-dihydroxyphenyl and 3,5-dimethoxyphenyl derivatives are better for tyrosinase inhibition than 2,5-dimethoxyphenyl derivatives. These results may be useful as a basis for the further synthesis of tyrosinase inhibitors.

### 3.11. Anti-Ulcerative Colitis Activities

**Figure 27:**
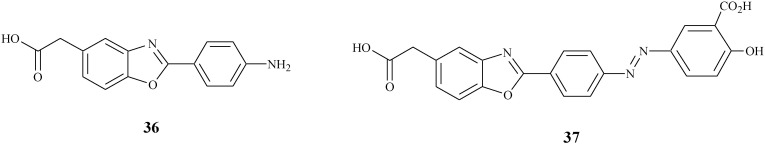
Chemical structures of aminobenzoxazole **36** and its azobenzene derivative **37**.

5-ASA has been widely used for the treatment of various diseases such as IBD, UC, and Crohn’s disease (CD), but one of the more recent purposes is to be able to reduce the side effects of the compound [53]. Jilani et al. reported the synthesis of an analog of a known nonsteroidal anti-inflammatory drug [NSAID], 4-aminophenylbenzoxazol-2-yl-5-acetic acid (**36**), considered a good anti-ulcerative colitis compound, and 5-[4-(benzoxazol-2-yl-5-acetic acid)phenylazo]-2-hydroxybenzoic acid (**37**) (a novel mutual azo prodrug of 5-aminosalicylic acid [5-ASA]) (Figure 27) [54]. Azo compound **37** and 4-aminophenylbenzoxazol-2-yl-5-acetic acid (**36**) were evaluated for trinitrobenzenesulfonic acid (TNB)-induced colitis in rats. The results demonstrated that the prepared azo prodrug was stable under acidic conditions similar to those of the upper gastrointestinal tract, which ensured its ability to reach the lower intestine intact. From the in vitro study, it was reported that bacterial azo reductase acted on the azo prodrug, and it was possible to correlate this with the capacity of the bacteria within the large intestine to digest the azo component, leading to a release of the 5-ASA and aminobenzoxazole.

Concerning diazo compound **37**, results showed that it had similar activity to 4-aminophenylbenzoxazole acetic acid compound **36** and 5-ASA. The similar potency between diazo compound **36** and both 5-ASA and **36** could be attributed to poor solubility and/or incomplete diazo reduction in the large intestine. However, since the absorption of this compound was expected to be low, it could represent a promising colon delivery system for both 5-ASA and aminobenzoxazole **36**. Synthesized diazo compound **37** and 4-aminophenylbenzoxazol-2-yl-5-acetic acid (**36**) were found to be as effective as 5-ASA for ulcerative colitis.

### 3.12. PPAR Ligands

**Figure 28:**
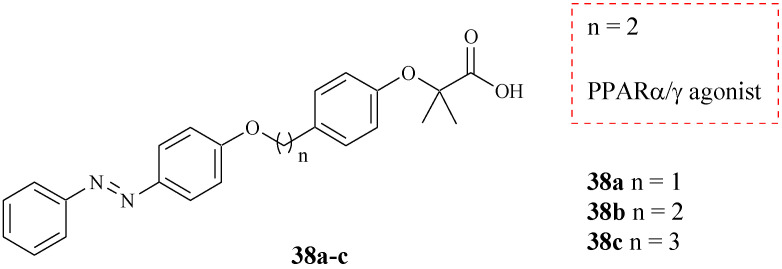
Chemical structures of azo compounds **38a**–**c**.

With the aim of developing compounds for the treatment of metabolic disorders, molecules based on a combination of fibric acid [55] and lipophilic groups derived from natural products such as chalcone and stilbene were synthesized [56]. In particular, some azobenzene derivatives (**38a**–**c**) were found to be active at micromolar concentrations as dual agonists PPARα/γ (Peroxisome Proliferator–Activated Receptors α/γ) (Figure 28).

The replacement of the double bond of stilbene with a diazenyl function gave a considerable increase in activity against PPARα, especially for compound **38b** (linker of two methylenes), which was also an activator of PPARγ (PPARα EC_50_ = 0.6 μM, PPARγ EC_50_ = 1.4 μM); this molecule probably fit better into the receptor pockets than the other two analogs (**38a** PPARα EC_50_ = 5.6 ± 0.3 μM, PPARγ EC_50_ = 21.0 ± 0.9 μM; **38c** PPARα EC_50_ = 42.8 ± 0.4 μM, EC_50_ = 10.0 ± 0.7 μM) [57].

**Figure 29:**
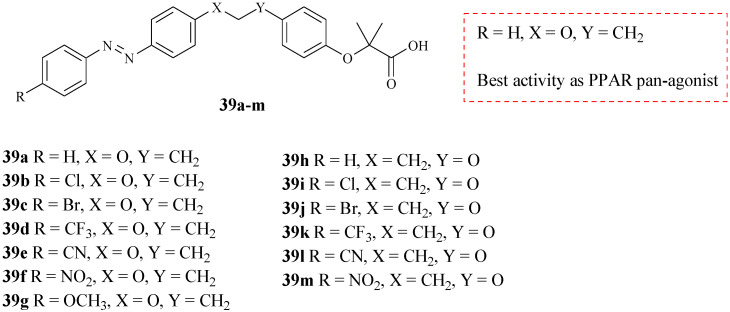
Chemical structures of azo compounds **39a**–**m**.

Starting from these results, two series of new phenyldiazenyl fibrate derivatives of PPARα/γ dual agonist **38b** were synthesized and tested (**39a**–**m**) (Figure 29) [58]. Compound **39a** (R = H, X = CH_2_, Y = O) showed balanced activity on the three PPAR isoforms α, γ, and δ with EC_50_ = 0.25 μM, 6.0 μM, and 2.8 μM, respectively, and, for this reason, **39a** was identified as a PPAR pan-agonist.

**Figure 30:**
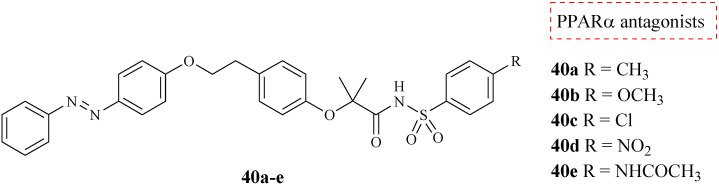
Chemical structures of azobenzenes **40a**–**e**.

Continuing this study, Ammazzalorso et al. synthesized some **38b** derivatives bearing the sulfonimide functional group to switch the activity from PPAR agonists to PPAR antagonists [59]. The bioisosteric replacement of the carboxylic group with a sulfonimidic one is a useful strategy in drug discovery because the sulfonimidic moiety shows a very similar profile to the carboxylic group in terms of acidity and H bond properties [60]. Five of them, reported in Figure 30 (**40a**–**e**), were tested on PPARα and PPARγ and showed IC_50_ values *versus* PPARα of 0.17 ± 0.12 μM, 0.33 ± 0.14 μM, 0.21 ± 0.13 μM, 1.1 ± 0.7 μM, and 1.5 ± 0.5 μM, respectively, and were considered novel PPARα antagonists.

**Figure 31:**
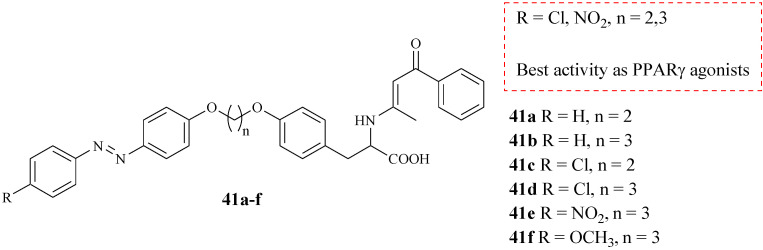
Chemical structures of compounds **41a**–**f**.

With the aim of discovering new PPAR agonists, De Filippis et al. synthesized tyrosine derivatives (**41a**–**f**) based on the combination of GW409544, a potent agonist of both PPARα and PPARγ, and a phenyldiazene scaffold (Figure 31) [61].

These molecules showed potent and selective PPARγ agonistic activity. Compounds **41c**–**e** with EC_50_ values of 0.039 ± 0.14 μM, 0.047 ± 0.012 μM, and 0.029 ± 0.07 μM, respectively, showed an activation of PPARγ comparable to the reference compound resveratrol (EC_50_ = 0.039 ± 0.003 μM), a potent and selective PPARγ agonist.

## 4. Conclusions

The azobenzene scaffold is studied in medicinal chemistry with the aim of developing novel agents implicated in a wide range of pathologies. This moiety is inserted in molecules to obtain anticolitic, antidiabetic, anti-inflammatory, antimicrobial, antiviral, anticancer, neuroprotective agents and compounds that act as enzymatic inhibitors or nuclear receptor ligands (Table 7).

**Figure 32:**
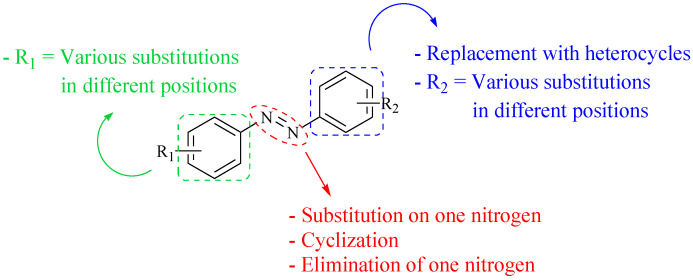
Chemical modifications of azobenzene scaffold.

In this review, the typical method for synthesizing azobenzene was described; moreover, it summarized the main pharmacological activities of these azo compounds that have been shown in recent years up until now. Furthermore, the chemical modification of the azo linker, substitution of aromatic rings, and replacement of one of the aromatic rings with heterocycles (Figure 32) were described in relation to the activity, with particular attention to SAR studies. It is not possible to obtain a single SAR analysis for all azobenzene derivatives summarized in this review, but each figure reports the importance of the chemical modification in reference to the biological activity. As a result, this work provides significant insight into azobenzene-containing compounds, with the aim of providing a useful tool in the design of novel azo-bearing molecules involved in various biological pathways through the combination of all modifications reported in this review.

## Figures and Tables

**Table 1:** Details of antimicrobial studies of most active tested compounds **6b**,**d**,**e**,**f**,**i**,**j**.

Compounds	Antibacterial Activity (Diameter of Zone Inhabitation in mm)				Antifungal Activity (Diameter of Zone Inhabitation in mm)			
	*E. coli*	*P. aeruginosa*	*K. pneumonia*	*S. aureus*	*C. albicans*	*A. flavus*	*A. fumigatus*	*T. rubrum*
**6b**	23	25	25	26	22	26	24	22
**6d**	14	17	19	16	15	16	20	18
**6e**	--	--	--	--	10	--	--	--
**6f**	--	14	--	--	--	12	--	09
**6i**	--	14	--	--	--	14	--	--
**6j**	12	16	15	14	13	08	11	11
**ciprofloxacin (Std)**	20	22	22	20	--	--	--	--
**ciclopiroxolamine (Std)**	--	--	--	--	20	22	22	20

‘--’ indicates that the compound was inactive.

**Table 2:** The antitumor activities of the tested compounds **20a**–**g** against MCF-7.

Compounds	R	R′	X	IC_50_ (μM)
**20a**	H	Me	H	6.49 ± 1.42
**20b**	H	Me	Me	1.35 ± 1.13
**20c**	H	Me	Cl	9.42 ± 2.20
**20d**	H	Ph	Me	15.37 ± 1.46
**20e**	Me	Me	H	3.71 ± 1.33
**20f**	Me	Me	Me	0.69 ± 1.10
**20g**	Me	Me	Cl	19.05 ± 1.84
**Doxorubicin**	--	--	--	0.44 ± 1.07

‘--’ indicates that the compound was inactive.

**Table 3:** Chemical structures of **22a**–**i** and complex **22g-CuCl_2_** and their anticancer activity against axenic cancer cells and cytotoxicity.

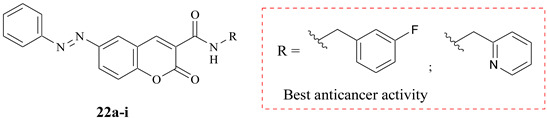						
Compounds	R	IC_50_ ± SE (μM)				
		HeLa	MCF-7	A549	HepG-2	HUVEC
**22a**	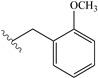	4.00 ± 0.06	>100	>100	2.50 ± 0.54	12.85 ± 0.56
**22b**	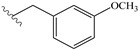	2.00 ± 0.37	1.05 ± 0.24	>100	1.70 ± 0.62	10.28 ± 0.92
**22c**	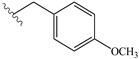	4.00 ± 0.90	3.03 ± 0.21	>100	3.61 ± 0.59	9.42 ± 0.74
**22d**	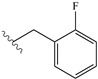	>100	>100	16.98 ± 1.32	>100	3.80 ± 0.66
**22e**	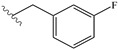	1.76 ± 0.31	>100	0.51 ± 0.22	1.59 ± 0.31	4.79 ± 0.91
**22f**	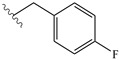	1.88 ± 0.83	>100	0.29 ± 0.20	0.51 ± 0.18	3.97 ± 0.75
**22g**	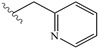	4.48 ± 0.43	0.42 ± 0.23	6.21 ± 0.86	6.05 ± 0.84	>100
**22h**	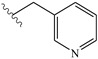	>100	6.44 ± 0.63	4.47 ± 0.60	24.15 ± 0.60	>100
**22i**	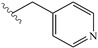	>100	17.32 ± 0.93	3.68 ± 0.28	3.62 ± 0.23	2.42 ± 0.59
**DOX**		1.34 ± 0.08	20.64 ± 3.67	1.18 ± 0.03	0.5 ± 0.18	1.6 ± 0.40

**Table 4:** *h*CA I and II inhibition data with sulphonamides **23**, **23a–f**, and clinically used inhibitor AZA and the selectivity ratio of *h*CA I to *h*CA II.

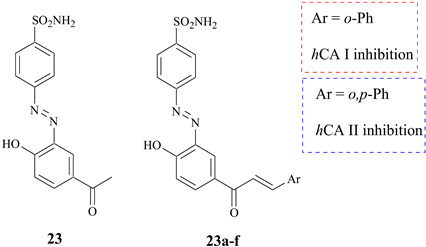				
Compounds	Ar	K_I_ (nM)		Selectivity Ratio (*h*CAII/*h*CAI)
		*h*CAI	*h*CAII	
**23**	**-**	19–10	42.30	2.21
**23a**	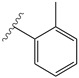	24.40	18.25	0.75
**23b**	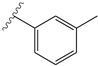	13.25	20.7	1.53
**23c**	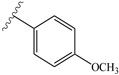	13.05	55.43	4.25
**23d**	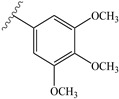	21.88	52.17	2.38
**23e**	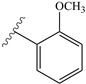	14.47	31.76	2.19
**23f**	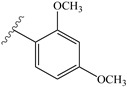	9.88	20.05	2.03
**AZA**	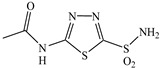	250	12.00	0.05

**Table 5:** Substitution pattern, inhibitory effects on mushroom tyrosinase, and kinetic analysis of active compounds **34a**–**d**.

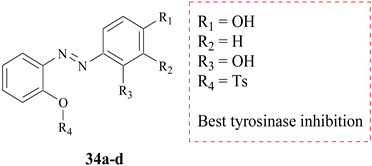						
Compounds	R_1_	R_2_	R_3_	R_4_	Tyrosinase Inhibition ** (%)	IC_50_ μM
**34a**	OH	H	H	* Ts	34.03 ± 8.52	200.77 ± 2.75
**34b**	OH	H	OH	Ts	88.84 ± 3.03	17.85 ± 0.17
**34c**	OH	H	H	H	82.50 ± 1.08	44.11 ± 0.88
**34d**	OH	H	OH	H	72.22 ± 1.08	48.08 ± 0.36
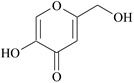 **kojic acid**	-	-	-	-	56.16 ± 0.85	49.08 ± 0.36
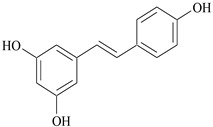 **resveratrol**	-	-	-	-	-	59.80 ± 0.98

Values indicate means ± SE for three determinations. * Ts: p-Toluenesulfonyl. ** Tyrosinase inhibition was measured using L-tyrosine as substrate at 50 μM.

**Table 6:** Tyrosinase inhibition and substitution pattern of substituted azo analogs **35a**–**i**.

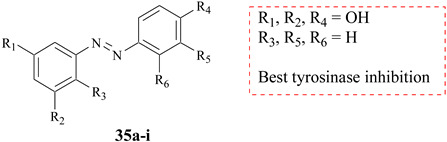							
Compounds	R_1_	R_2_	R_3_	R_4_	R_5_	R_6_	Tyrosinase Inhibition * (%)
**35a**	OMe	OMe	H	OH	H	H	56.25 ± 0.45
**35b**	OH	OH	H	OH	H	H	72.75 ± 1.70
**35c**	OMe	OMe	H	OH	H	OMe	8.90 ± 1.87
**35d**	OMe	OMe	H	OH	OMe	H	1.03 ± 9.08
**35e**	OMe	OMe	H	OH	H	OH	48.50 ± 3.47
**35f**	OH	OH	H	OH	H	OH	41.46 ± 2.17
**35g**	OMe	H	OMe	OH	H	OH	27.80 ± 1.14
**35h**	OMe	H	OMe	OMe	H	OH	6.38 ± 2.79
**35i**	OMe	H	OMe	OH	OMe	H	0.44 ± 1.65

Values represent means ± S.E. of three experiments. * Tyrosinase inhibition was measured using L-tyrosinase as the substrate at 50 μM.

**Table 7:** Summary of the general structures of the most representative azobenzene derivatives and their main activities.

Compounds	Structures	Pharmacological Effects	References
**1**	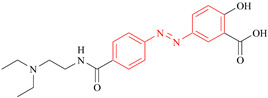	Anticolitic	[6]
**2**	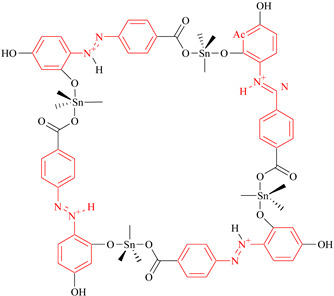	Antidiabetic	[10]
**3a**–**j**	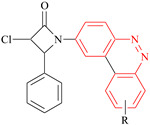	Anti-inflammatory	[11]
**4a**–**j**	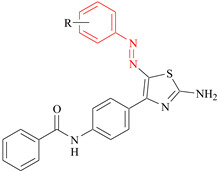	Antimicrobial	[12]
**5a**–**c**	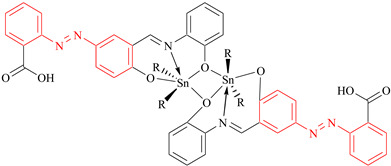	Antibacterial Antifungal	[13]
**6a**–**j**	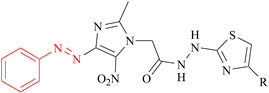	Antibacterial	[14]
**7a**–**e**	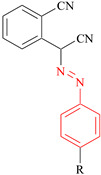	Antibacterial Antifungal	[15]
**8a**–**d**	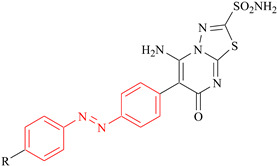	Antibacterial Antifungal	[16]
**9a**–**o**	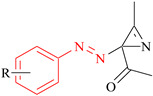	Antimicrobial	[17]
**10a**–**p**	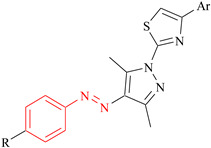	Antifungal	[18]
**11a**–**u**	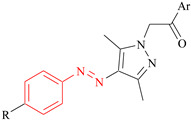	Antibacterial Antifungal	[19]
**12**	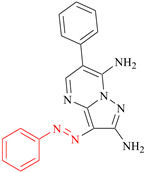	Antimicrobial	[20]
**13a**–**g**	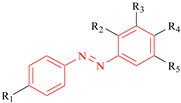	Antimicrobial	[21]
**14a**–**i**	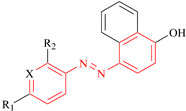	Antimicrobial	[24]
**15**	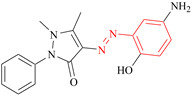	Antimicrobial	[25]
**16**	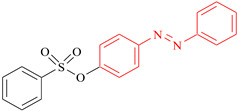	Antimicrobial Antifungal	[29]
**17a**–**k**	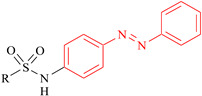	Anticancer	[30]
**18a**,**c**,**f**	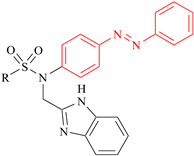	Anticancer	[30]
**19a**–**l**	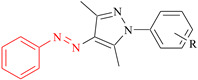	Anticancer	[32]
**20a**–**g**	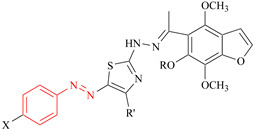	Anti-breast cancer	[33]
**21a**–**c**	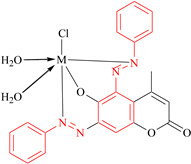	Anti-breast cancer	[34]
**22a**–**1**	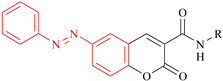	Anticancer	[35]
**23a**–**f**	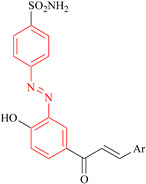	CA inhibitors	[36]
**24a**–**e**	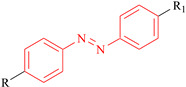	CA inhibitors	[37]
**25a**–**e**	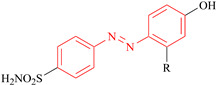	HpCAs inhibitors	[38]
**25a**–**l**	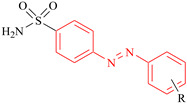	COX-2 inhibitors	[41]
**27a**–**c**, **28**	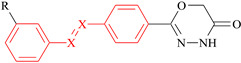	Anti-MAO-B	[42]
**30a**–**b**	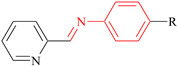	Anti-MAO-B	[42]
**30a**–**c**	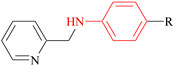	Anti-MAO-B	[42]
**31a**–**b**	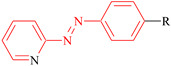	Anti-MAO-B	[43]
**32**	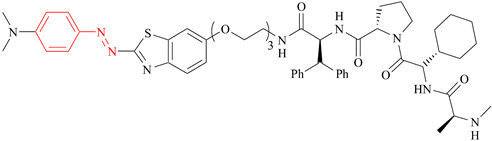	Neuroprotective	[45]
**33a**–**c**	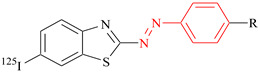	Neuroprotective	[48]
**34a**–**d**	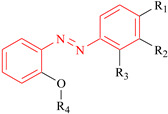	Tyrosinase inhibitors	[50]
**35a**–**i**	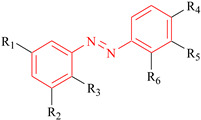	Tyrosinase inhibitors	[53]
**37**	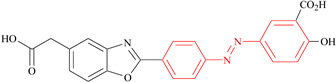	Anti-ulcerative colitis	[55]
**38a**–**c**	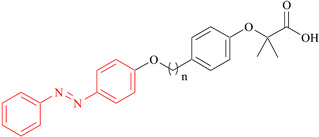	PPARα/γ agonists	[57]
**39a**–**m**	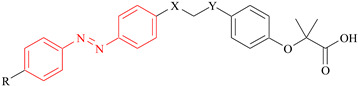	PPAR pan agonists	[58]
**40a**–**e**	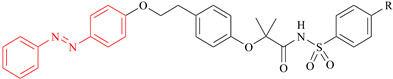	PPARα antagonists	[60]
**41a**–**f**	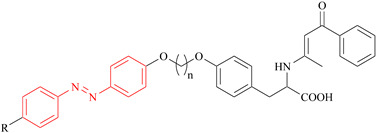	PPARγ agonists	[61]

## Data Availability

No new data were created or analyzed in this study.
